# Exploring Technology Integration in Health and Safety Routines in a Shanghai Kindergarten

**DOI:** 10.3390/healthcare13030218

**Published:** 2025-01-22

**Authors:** Wenwei Luo, Xiaoyu Wu, Ilene R. Berson, Michael J. Berson, Huihua He, Minqi Gao

**Affiliations:** 1 Shanghai Institute of Early Childhood Education, Shanghai Normal University, Room 507, Jiaoyuan Building, 100 Guilin Road, Shanghai 200234, China; wenweiluo@shnu.edu.cn (W.L.);; 2 Lab for Educational Big Data and Policymaking, Ministry of Education, Shanghai 200234, China; 3 Marsal Family School of Education, University of Michigan, Ann Arbor, MI 48109, USA; wuxiaoyu@umich.edu; 4 Department of Teaching and Learning, College of Education, University of South Florida, Tampa, FL 33620, USA; iberson@usf.edu (I.R.B.); berson@usf.edu (M.J.B.)

**Keywords:** digital health technologies, early childhood education, health and safety routines, Shanghai kindergarten, qualitative research

## Abstract

**Background**: Digital technology is increasingly being used in early childhood education; however, there is a significant gap in understanding how these technologies are practiced in health and safety routines. **Objective**: This study aims to understand the role of technologies in daily health and safety checks and identify the issues arising from their use in a Shanghai kindergarten. **Method**: A qualitative approach was employed; the study involved video analysis and semi-structured interviews with 8 teachers, 18 parents, 11 children, and 3 principals from a leading kindergarten in Shanghai. Data were analyzed using NVivo 12 to uncover themes related to technology use, human–technology interactions, and operational challenges. **Results**: The findings show that digital technologies enhance operational efficiency, but they also present challenges like usability issues and technical limitations. The study underscores the critical need for more child-friendly and educator-accessible designs to maximize the potential of these technologies. **Conclusions**: This study highlights the transformative potential of digital technologies in kindergarten health and safety routines. This insight contributes to a broader discourse on the benefits and complexities of digital transformation in early childhood education. Future research should focus on scalable, inclusive solutions and enhanced data governance to maximize the benefits of those tools in diverse educational settings.

## 1. Introduction

Prior research has extensively explored the integration of technology into early childhood education and care (ECEC), examining various profiles and practices [1,2,3]. While these studies often focus on the frequency and strategies for applying digital devices within ECEC settings, a growing body of research highlights digital pedagogical practices, including the use of artificial intelligence (AI), computational thinking (CT), programming education (both plugged and unplugged), and digital learning and play across diverse preschool and kindergarten contexts [4,5,6].

The perceptions and values of various stakeholders—children, teachers, school administrators, and parents—toward these digital developments are varied and often complex [7,8]. These stakeholders frequently debate the roles technology plays in human–technology interactions, particularly in terms of its potential to support or hinder young children’s development [9]. Additionally, stakeholders face a variety of needs and challenges. For instance, while teachers and parents acknowledge the convenience of technological integration, they also encounter significant obstacles. These discussions underscore the importance of educators’ pedagogical digital literacy, including their ability to assess the benefits of digital tools, effectively utilize and teach with digital devices, and remain vigilant about potential risks to children [10,11].

Despite advancements in descriptive and empirical research, limited attention has been paid to the role of technology in supporting children’s hygiene, healthcare, and safety. Studies and global policy reports emphasize that high-quality ECEC services should adopt a holistic and inclusive approach, integrating learning, nurturing, child-rearing, and social support for young children [12,13]. These approaches conceptualize “care” and “education” as interconnected components of quality ECEC services [14,15].

Shanghai kindergartens operate under the guidance of the Shanghai Municipal Commission of Education, which is responsible for upholding educational standards, implementing safety protocols, and ensuring a consistent and high-quality learning environment [16]. Health and safety routines within Shanghai’s ECE system are carefully designed to safeguard children’s well-being, including safety management, emergency response protocols, regular safety education, and training sessions, aimed at promoting both physical and emotional health for children [17]. With advancements in technology, modern tools are increasingly integrated into healthcare and safety procedures, becoming an integral part of daily operations. This integration involves multiple stakeholders, including children, parents, and teachers, and presents a valuable opportunity to expand research on digitalization in ECEC [18]. Investigating how technology is integrated into healthcare and safety scenarios in kindergartens can deepen understanding of digital practices in early learning environments and provide insights into the broader implications of technological adoption in ECEC.

This study aims to address two central research questions: (1) How are health and safety routines in Shanghai kindergartens conducted in alignment with digital technology integration? (2) What problems have been resolved, and what new challenges and issues have arisen in the context of the growing digitalization of the ECEC field?

### 1.1. Technology Integration in ECEC Settings

The integration of appropriate technology in ECEC plays a crucial role in improving educational quality and fostering children’s development across multiple domains. As a key component of digitalization in early childhood education, technology supports a wide range of educational applications, offering tools to enhance teaching and learning effectiveness. Prior research has extensively examined these scenarios by exploring the forms and functions of technology and evaluating the outcomes of technology-based interventions. For example, Colliver et al. [10] found that technology could enhance young children’s learning, exploration, and research. Additionally, ECE institutions have leveraged digital platforms for planning, observation, assessment, and the development of child portfolios [19,20]. These platforms also allow teachers to include critical reflections, fostering a culture of continuous improvement.

Family engagement is another vital area of focus. Social media technologies enable schools to share information about operations, activities, upcoming events, and curricula with families, allowing parents to review and provide feedback [21,22]. Furthermore, technology facilitates professional development for educators. Teachers can participate in webinars tailored to their needs and interests, collaborate with colleagues, and engage in professional conversations, which can positively impact their practice and school leadership [3,23].

While empirical studies affirm the value of technology in enhancing human–technology interaction and supporting integrated teaching and learning, they also highlight challenges. These include the development of hardware infrastructure, varying stakeholder perceptions, risk protection, ethical considerations, and the accurate use of data. For example, Luo et al. [24] conducted a systematic review on the readiness of Chinese preschool teachers to integrate technology in their classrooms. The findings revealed that many teachers lack adequate preparation for optimizing the use of digital tools. Addressing gaps in teachers’ digital literacy—such as technological skills, attitudes toward appropriate technology use with young children, and an understanding of digital pedagogy—is crucial for advancing technology integration in ECE settings [25,26]. Moreover, the availability of context-specific, child-friendly devices, robust school networks, and adequate hardware infrastructure significantly influences the experiences of children and educators [27].

Despite the breadth of research on technology integration across various scenarios, certain contexts remain underexplored. For example, the use of technology for monitoring children’s health data or supporting the overall operation and management of kindergartens has received little empirical attention. These areas, while not directly tied to traditional teaching and learning activities, can indirectly impact children’s development and educational experiences. Further investigation into these less commonly explored scenarios is necessary to provide a more comprehensive understanding of technology’s role in ECEC settings.

### 1.2. Safety and Healthcare Routines Supported by Technology

While the educational impact of technology in safety and healthcare routines may be subtle and often overlooked, its practical applications have long been underway, addressing critical challenges in kindergarten management—particularly the shortage of healthcare personnel. In this context, technology has become essential for performing tasks such as registration, health checks, and identity verification at entrances. For example, technologies like facial recognition for identifying children, automated measurements of height and weight, and verification of personal information streamline these processes. This not only enhances operational efficiency but also alleviates the burden on limited healthcare staff in kindergartens.

The use of digital devices has emerged as a compensatory solution for the scarcity of healthcare personnel, significantly improving productivity and efficiency in early childhood settings. Knauer et al. [28] highlighted the resource constraints faced by schools in meeting students’ health needs, noting that school nurses and healthcare staff often struggle to provide adequate care. Similarly, school-based health centers (SBHCs) face resource shortages that limit their ability to offer comprehensive healthcare services to students [29]. The issue of healthcare personnel shortages became even more pronounced during the COVID-19 pandemic [30]. Despite these challenges, healthcare staff in kindergartens remain responsible for substantial workloads, including immunizations and general health promotion, which place significant demands on their capacity [31,32].

Beyond addressing staff shortages, technology enhances data management and decision-making. Digital devices can record and store health data in backend databases, enabling easy retrieval and analysis of individual and group information over specific periods. This facilitates targeted interpretation and early detection of special health needs or potential issues. Traditional morning health checks, which are often repetitive and passive, can sometimes lead to resistance from children. However, when these checks are conducted with the assistance of robots, children show increased engagement, a stronger willingness to participate, and a sense of accomplishment, potentially reducing resistance to the routine.

Despite the increasing presence of technology in school safety and healthcare practices, this area remains underexplored in empirical research. Investigating these practices can shed light on their processes, validate their necessity and feasibility, and clarify the roles of various stakeholders—including technology, children, parents, and teachers—in operational workflows. Furthermore, such research can identify the challenges and issues that arise, paving the way for more effective and inclusive technological integration in early childhood safety and healthcare.

### 1.3. Theoretical Framework: Actor–Network Theory

Actor–network theory (ANT) provides a powerful theoretical framework for analyzing the dynamics of complex systems by emphasizing the interactions between human and non-human actors within a network [33]. Rather than attributing causality to any single actor or factor, ANT views all actors as interconnected elements that collectively influence and are influenced by one another. This perspective sheds light on how interactions among these elements contribute to the formation, evolution, and stability of social phenomena.

In the context of implementing new educational technologies, ANT offers valuable insights into how teachers, students, administrators, and the technology itself form networks that shape the adoption and effectiveness of these tools [34,35]. For example, an ANT approach might reveal how a digital learning platform reshapes teaching practices and student engagement by examining the interplay between the platform’s design, teachers’ instructional strategies, and students’ responses. This networked perspective can explain variations in how different schools experience and benefit from the technology.

Similarly, in healthcare settings, ANT can elucidate how networks of doctors, nurses, patients, and electronic health record (EHR) systems interact to influence the quality of care [36]. The theory can demonstrate how the introduction of EHRs alters workflows, communication patterns, and decision-making processes, emphasizing that the system’s success relies on the alignment and negotiation among all actors in the network.

Applying ANT to early childhood education (ECE) extends our understanding of technology by positioning it as an integral part of a larger network. By viewing technology as both a tool and an active participant, ECE institutions can reflect on its role in fostering innovation and enhancing service quality. However, challenges arise from the fragility of emergent pedagogical strategies, the requirements for teacher professional development, and the adequacy of technological infrastructure in schools. Through the lens of ANT, researchers can gain deep insights into the complex relationships and mechanisms driving change in ECE settings. This approach highlights how interconnected human and non-human actors collaboratively shape educational outcomes, offering a nuanced understanding of the forces that influence technology integration and its impact.

## 2. Methods

This study employed a triangulation strategy, combining qualitative methods and diverse data sources to ensure a comprehensive and credible investigation of the research questions. Data collection and analysis were conducted using video recordings and interviews, which allowed the study to capture different aspects of participant experiences. By integrating these methods, the study addressed the limitations of relying on a single approach, offering a detailed understanding of the research context.

Data were gathered from three participant groups: children, parents, and teachers (including principals, considered senior teachers). Comparing findings across these groups enabled cross-validation, revealing patterns of agreement as well as discrepancies that enriched the analysis with unique insights. This triangulation strategy also facilitated the identification of unexpected findings, enhancing the depth and credibility of the study [37]. Particularly effective in exploratory research, where the aim is to examine complex and understudied areas [38], this approach provided a strong framework for investigating the integration of technology in preschool healthcare routines.

### 2.1. Research Site and Participants

Shanghai is recognized as a leader in the integration of technology and instructional practices in early childhood education within China [39]. Kindergartens in Shanghai are systematically categorized into four tiers: Model-level, Level 1, Level 2, and Level 3, with model-level kindergartens excelling across all domains [40,41]. For this study, we selected a model-level kindergarten centrally located in Shanghai. This institution serves as an exemplar for digital technology integration in early childhood education, supported by substantial annual funding and equipped with state-of-the-art digital resources designed to enhance teaching, healthcare, home–school collaboration, and other educational activities.

We employed a combination of purposeful sampling and convenience sampling strategies to select participants. Purposeful sampling [42] ensured the inclusion of individuals directly involved in the kindergarten’s health and safety routines, such as teachers, parents, and children. This approach allowed us to focus on participants with direct experience and relevant insights into the study’s area of interest, enhancing the depth and relevance of the data collected. Convenience sampling [43] was then used to facilitate access to participants who were readily available and willing to participate in the study. The final cohort included 40 participants: 8 teachers, 18 parents, 11 children, and 3 principals. Detailed demographic information is provided in Table 1, Table 2 and Table 3. To maintain clarity, participants’ roles were represented by initials—T for Teacher, P for Parent, and C for Child—which were used consistently throughout the study. Demographic characteristics, including gender, age, and professional background, were also considered during recruitment to ensure a diverse and representative sample, providing a broader range of perspectives.

Our study focused on health and safety routines within kindergartens, an area distinct from other aspects of early childhood education. To capture a range of perspectives, we employed a multi-agent analysis approach, gathering viewpoints from various stakeholders. Conducting interviews across all participant groups allowed us to explore the complexity of healthcare routines in kindergartens, ensuring that the diverse roles and experiences of those involved were represented. This approach enhanced the credibility of our findings and offered valuable insights into the dynamics of kindergarten healthcare practices.

### 2.2. Data Collection

This study employed a two-phase approach to examine the role of digital technologies in healthcare services within the kindergarten context, focusing on challenges addressed and emerging issues. The first phase documented the digital infrastructure through video recordings, capturing its application in health and safety routines. In the second phase, semi-structured interviews were conducted with parents, children, teachers, and principals to explore their perspectives on the integration and functionality of digital technology in kindergarten healthcare practices.

### 2.3. Video Data Collection

The video recordings documented the integration of digital technology into kindergarten health and safety routines over a period of 2–3 months. This phase focused on two key technological tools: the smart attendance device and the morning inspection robot. Four video recordings were collected during this period, with each video ranging from 8 to 11 min in length. A dual-camera setup allowed for comprehensive data capture, with one camera positioned to provide a wide-angle view of the overall environment and the other focused on close-up interactions between children, parents, teachers, and the technology. This configuration facilitated the simultaneous observation of group dynamics and individual behaviors, capturing verbal and non-verbal communication from multiple perspectives. The total duration of video data analyzed amounted to approximately 40 min.

### 2.4. Semi-Structured Interview

Following video data collection, we conducted individual semi-structured interviews to explore stakeholder perspectives. These interviews sought to uncover how digital technologies addressed existing challenges in kindergarten healthcare routines and to identify emerging needs. Given the differing roles and experiences of teachers, principals, parents, and children, the interviews provided a deeper understanding of the perceived value, functionality, and limitations of technology integration in this context.

Interviews were conducted in quiet, familiar settings to enhance participant comfort and encourage open communication. For young children, interviews occurred during instructional breaks in their classroom setting, which helped to promote a sense of security and foster candid responses. Participation was voluntary, and only children who willingly agreed to participate were included. A teacher was present during the interviews to provide support and clarify questions as necessary, ensuring that the children fully understood the questions and could contribute thoughtful and relevant perspectives on their experiences.

Open-ended questions encouraged participants to share diverse and detailed responses. Interviews with parents and children lasted between 10 and 30 min, while interviews with teachers and principals averaged over 45 min, allowing for more extensive insights. All interviews were recorded and transcribed verbatim in Mandarin, ensuring fidelity to the original data. The transcripts were carefully analyzed to capture themes relevant to the research questions.

Ethical clearance for this study was granted by the first author’s affiliated university (IRB Number: 2023046), and pseudonyms have been used to ensure anonymity.

### 2.5. Data Analysis

This study utilized content analysis [44], combining deductive and inductive approaches to analyze video and interview data using Nvivo12. The analysis examined how technology and humans (children, parents, teachers, and principals) transitioned between roles, guided by the principles of actor–network theory (ANT). Role transitions refer to the dynamic shifts in how technology and individuals interact and fulfill different functions within kindergarten healthcare routines, adapting to specific tasks and contexts. For example, technology such as the smart attendance device may function as an independent actor by autonomously recording attendance and then transition to a facilitator when its data supports teacher decision-making. Similarly, human actors, such as a teacher, may act as an independent actor while overseeing a health check but shift to a responsive entity when adapting to data generated by a morning inspection robot. These transitions reflect the interconnected and evolving interactions between human and technological actors, shaping healthcare practices in the kindergarten setting.

The video data analysis began with a deductive approach, which provided a structured framework for identifying roles based on ANT concepts (e.g., independent actor, facilitator, responsive entity). An inductive approach followed, allowing additional themes, categories, and codes to emerge organically from the data. Temporal coding was applied to examine patterns such as the frequency and duration of specific interactions, offering insights into recurring behaviors. Multiple viewings of the recordings also enabled the extraction of representative details—such as brief events, images, and conversations—that enriched the thematic analysis. These elements contributed to a comprehensive categorization of roles for technology and humans, exploration of interaction dynamics, and examination of environmental and contextual factors influencing technology use and perception.

The interview data analysis similarly employed an inductive coding method. Initial descriptive codes summarized key data segments, forming the basis for higher-order coding and thematic development [45]. Prominent themes emerged through iterative reviews, with insights grouped into broader, abstract categories. Examples of the coding process and the final coding scheme are presented in Table 4 and Table 5.

The analysis of both video and interview data incorporated NVivo 12 to organize and refine themes, using its word frequency and cluster analysis tools to identify patterns, relationships, and prominent themes within each dataset. This facilitated systematic coding and supported the integration of findings across the two data sources. To ensure the credibility and reliability of the analysis, the research team conducted peer debriefing sessions [46,47], which refined the coding and thematic development, enhancing consistency and accuracy. Additionally, an inquiry audit by the author team confirmed that the results aligned with the study’s research objectives and accurately reflected the data. This iterative process resulted in a final coding scheme with strong inter-coder agreement.

## 3. Findings

### 3.1. Technology in Kindergarten Health and Safety Practices

#### 3.1.1. Smart Attendance System

The smart attendance device serves as a technological safety tool designed to facilitate the secure and efficient daily entry of children into the kindergarten, as illustrated in Table 6. The system consists of a gate and a small screen where parents or children scan a QR code. The device verifies essential information, including the child’s name, class, and COVID-19 test results—features particularly critical during the pandemic. Children meeting the health and safety criteria (e.g., confirmed identity, no symptoms of illness, and a negative COVID-19 test) are granted entry, with the gate opening automatically upon successful verification. This system streamlines the entry process while reinforcing safety measures.

As shown in Table 7, video analysis of the smart attendance device affirmed that it was designed to operate as an Independent Actor, autonomously verifying entry criteria without facilitating interactive roles for parents or children. Its primary function as an objective screener ensures impartiality but inherently limits user engagement. In this role, the device prioritizes efficiency, relegating parents and children to passive observers during its operation. While this design achieves its safety and efficiency objectives, it introduces limitations in user experience by neglecting opportunities for interactivity, which may reduce satisfaction and engagement among families.

Our analysis indicated that interpersonal interactions, such as communication between parents, children, and school staff, were more critical to the success of the system than interactions between humans and the technology itself. Challenges, such as low sensitivity or Wi-Fi delays, were largely resolved through direct communication with school personnel rather than through technological adjustments. Specifically, 10.53% of parents who encountered operational issues relied on teachers and staff for assistance.

In contrast, approximately 89.47% of parents demonstrated autonomy in operating the system without the need for external assistance, which highlighted the simplicity and ease of use of the device. However, the device lacked features to provide direct guidance to children, a gap observed during its use. In these instances, parents often assumed the role of a facilitator, guiding their children through the process—a role the technology itself could potentially have fulfilled. This finding highlighted the limited scope of the human–technology interaction, as the device predominantly functioned as an Independent Actor, restricting the depth of engagement between users and the system.

In summary, the smart attendance device effectively fulfills its safety-oriented purpose, offering a simple and efficient design that enables parents to operate it independently. However, its focus on simplicity comes at the cost of broader functionality and interactivity. The device’s limited capacity to engage children directly or address diverse user needs leaves parents to compensate as facilitators, revealing an opportunity for future enhancements to balance efficiency with richer interaction capabilities.

#### 3.1.2. Morning Inspection Robot

The morning inspection robot conducts daily health checks on kindergarten children using a combination of a screen, scale, and camera to assess and display health details, as shown in Table 8. The robot first identifies children through facial recognition and then examines physical features such as the mouth and hands. It audibly announces results and displays photos on its screen, collecting data on height, weight, body temperature, and the condition of teeth, nails, and hands. This system contributes to the systematic collection of health data and supports efforts to monitor children’s well-being within the kindergarten setting.

As shown in Table 9, interactions with the morning inspection robot illustrate dynamic role transitions between humans and technology. The robot transitions through roles as a Facilitator, Responsive Entity, and finally an Independent Actor, while humans shift between roles as Observers and Responsive Entities during the inspection process. At the start of the interaction, the robot autonomously uses its camera to recognize the child, announces their name, and verifies their identity. During this stage, the robot assumes the role of a Facilitator, guiding the process by issuing verbal instructions such as “Please open your mouth” or “Please pay attention to your nails,” while the child passively observes. As the inspection progresses, the robot adjusts its lighting and focus based on the child’s movements and surroundings, while the child actively participates by opening their mouth or raising their hands. In this phase, both the robot and the child engage as Responsive Entities, directly interacting with one another. Once the inspection is complete, the robot displays a photo and verbally communicates the results, transitioning into the role of an Independent Actor that operates without further human input, while the child reverts to being an Observer.

The analysis of interaction dynamics reveals key trends in children’s communication behaviors with the morning inspection robot. Notably, 28.58% of children refused to use the robot entirely, opting instead for traditional teacher-led checks. Among those who successfully engaged with the robot, 22.86% avoided verbal communication with the device. Active verbal exchanges between children and the robot were rare, with passive or no communication dominating most interactions. Passive communication involved minimal, non-verbal responses, such as following the robot’s instructions (e.g., opening their mouth or raising their hands) without speaking or actively engaging. In contrast, non-verbal communication, such as gestures and eye contact directed at the robot, was more prevalent and appeared to be a preferred mode of interaction for many children.

These findings suggest that children’s preference for non-verbal modes of communication may have multiple underlying causes. Non-verbal interactions might be perceived as more intuitive or less demanding compared to verbal exchanges. Alternatively, this behavior could reflect discomfort with the interaction itself, such as unease with the robot’s verbal prompts or its mechanical nature. Another potential factor is a reluctance to engage due to the public sharing of personal data, such as the audible announcement of health information. Further research is needed to explore these factors and better understand the reasons behind children’s communication preferences with the robot.

Although the morning inspection robot demonstrates more diverse role transitions compared to the smart attendance system, its interactions remain limited in depth. There were no observed cases where human actions or decisions meaningfully influenced how the technology operated or was perceived. Similarly, the robot showed no evidence of adapting or responding to human actions. Verbal engagement with the robot was minimal, and the interaction was primarily cooperative, lacking reciprocal adaptation or responsiveness between the child and the technology. These limitations highlight opportunities to enhance the robot’s design to support richer and more responsive interactions with its users.

### 3.2. Barriers to Effective Technology Integration

#### 3.2.1. Infrastructure Challenges

The integration of technology in kindergarten settings has enhanced autonomous participation among parents and children. Parents have generally demonstrated proficiency in using the smart attendance device, and even when they encounter challenges, they persist in resolving them to successfully engage with the technology. Similarly, the morning inspection robot has been well-received by a majority of children (71.43%), with many eagerly anticipating their turn to use it. Table 8 illustrates children’s excitement while lining up to interact with the robot.

Despite this enthusiasm, inadequate infrastructure presents significant challenges, particularly in terms of accuracy, sensitivity, and network connectivity.

**Technical Limitations.** While labeled as “smart”, the digital devices in use frequently exhibit issues with accuracy and sensitivity. More than half of the participants highlighted concerns regarding accuracy, which can hinder productivity and user satisfaction. Participant P2 explained, “The morning check robot’s camera captures the entire mouth of the child but struggles to interpret the observed data”. Similarly, P9 emphasized, “Robotic inspections may lack precision, with teacher inspections being more detailed”.

Sensitivity was another significant concern. The smart attendance device often responds slowly, creating delays during entry and exit. Participant P17 noted, “Smart attendance devices might fail to recognize QR codes, causing morning entry queues”. Observations revealed instances where children stood in front of the morning inspection robot only to experience recognition failures, preventing the completion of health checks. Participant C9 echoed these frustrations, stating, “Both smart attendance devices and morning inspection robots sometimes fail to recognize me”.

These technical limitations not only reduce operational effectiveness but also introduce additional administrative burdens. For example, failures in data synchronization between devices and the school system can create inefficiencies and foster frustration among educators and students alike. This highlights the pressing need for improvements in the accuracy and sensitivity of these devices to ensure reliable and seamless operations. To address these limitations, developers may focus on integrating advanced error-detection algorithms and improving device calibration to enhance sensitivity and precision.

**Network Connectivity Issues.** The functionality of technological devices in kindergartens is heavily reliant on stable network connectivity, yet this remains a notable limitation. Participant P15 stated, “Network circumstances constrain smart attendance devices, and if the network signal is not linked, this device cannot be used, which is annoying”. Similarly, P14 described, “The network at the kindergarten’s entrance is occasionally weak, and the QR code for entrance on the cell phone does not display”. These network issues disrupt device functionality and hinder smooth operations, as the devices are unable to process essential data without a reliable signal.

Teachers also identified network constraints as a barrier to efficient use of these systems. Participant T4 remarked, “The convenience of smart attendance devices is contingent on network connectivity; if the signal is absent, the device becomes nonfunctional”. This feedback underscores the necessity of robust networks to support the effective operation of these digital devices in kindergarten settings. Implementation of stronger network infrastructure, such as dedicated Wi-Fi access points or backup connections, may mitigate this issue. Additionally, embedding offline functionality in devices could allow them to operate temporarily without a network, reducing dependency on real-time connectivity.

**Inefficient Processes.** In addition to network connectivity problems, inefficiencies in the photo-uploading process for the morning inspection robot further exemplify the lack of a fully prepared technological environment. Participant T8 described this process as overly reliant on a multi-step system requiring the involvement of an information supervisor:

These robots’ face recognition requires photo uploading, but the current photosystem requires the information supervisor to operate, which means that the teachers need first ask parents for images and then hand them over to the information supervisor. This process is time-consuming, and only the information manager will know if the child’s photo was successfully imported. It would be convenient if the teacher could manage the photo upload. At the same time, during the import process, there are frequently unsuccessful cases that necessitate restarting the machine and repeating the import processes until they are properly completed.

The reliance on a centralized system for photo uploads delays the functionality of the robot and increases the workload for teachers and supervisors. Simplifying the upload process and allowing teachers to independently manage photo uploads could reduce bottlenecks and enable smoother operations.

#### 3.2.2. Challenges in User Engagement and System Design

**Technology Needs to Be User-Friendly**. The alignment of technology with user preferences and knowledge is critical in educational and healthcare settings, where diverse stakeholders—including children, teachers, and families—engage with digital tools. Children’s reactions to technology, for instance, vary widely, reflecting the challenge of designing child-friendly systems. Some children, like C2, expressed enthusiasm for interactive and visually engaging features, stating, “I like robots because there are many buttons and QR codes on the robots, which are fun”. However, others held less favorable views. Child C5 described the morning inspection robot as intimidating: “The morning inspection robot looks a bit scary. It would be great if it could resemble Elsa and engage in interactive games with me”. Similarly, C11 suggested a more personal touch, saying, “I wish the robot could have two arms so it could hug me”.

These diverse reactions highlight the importance of designing technology that accommodates varying preferences and fosters a sense of comfort and engagement. During observations of the morning inspection robot, approximately one-third of the children exhibited reluctance to interact with the device, underscoring the need for designs that prioritize user-friendliness and appeal to children’s developmental needs.

In addition to visual and interactive elements, ergonomic factors significantly impact usability. Participant T3 noted, “If a child is under 1 m tall, the morning inspection robot’s camera cannot capture them, rendering the child unable to utilize the morning inspection robot”. This height limitation similarly affects the smart attendance device, as described by C8: “The smart attendance device is also too high. Younger children can’t reach it if they want to use it”. Observations further revealed instances where children struggled to use the attendance device without parental assistance, emphasizing the importance of designing systems that are physically accessible to young users. As Participant C8 suggested, shorter designs could better accommodate younger siblings and ensure independent use by all children.

Aligning technology with users’ knowledge levels is also essential, particularly when involving families of diverse ages. While many parents embrace the integration of healthcare technology, older family members, such as grandparents, often find the systems challenging to navigate. Participant P10 expressed, “The technology is too intricate, and we are too old to acquire the knowledge to operate them”. This reluctance highlights the gap in digital literacy among older users, which may hinder widespread adoption and acceptance of these tools.

**Training and Support for Effective Use.** Educators echoed similar concerns, emphasizing the need for training to maximize the potential of these technological systems. Participant T4 explained, “We often rely on trial-and-error to navigate these devices because there isn’t enough formal training provided. If we had clearer guidance, we could use these systems more effectively”. Without sufficient training, educators are burdened with troubleshooting and learning through experience, which detracts from their primary responsibilities and reduces the overall efficiency of technology integration.

To address these challenges, educational institutions and healthcare providers must prioritize the development of user-friendly interfaces and offer comprehensive training programs tailored to diverse audiences. These programs should account for the needs of children, teachers, and family members, particularly grandparents, to bridge the technological knowledge gap and foster greater acceptance of digital advancements. Ensuring that technology is both accessible and intuitive can significantly enhance user engagement and streamline its integration into early childhood education and healthcare settings. By addressing usability concerns through thoughtful design and tailored training, these systems can better meet the needs of all stakeholders, ensuring that healthcare technology fulfills its potential to support children’s development and well-being in a collaborative and inclusive manner.

### 3.3. Impacts of Technology on Kindergarten Practices

#### 3.3.1. Improved Operational Efficiency

The integration of healthcare technology in early childhood settings has garnered widespread approval among both teachers and parents, who recognize its potential to address resource limitations and enhance efficiency. One of the key benefits highlighted is its ability to mitigate the shortage of healthcare staff. Participant T4 shared, “In our kindergarten, there’s only one healthcare personnel who conducts time-consuming morning check-ups for all the children. However, introducing a morning inspection robot is seen as effective in reducing her workload and compensating for the healthcare shortage”.

Parents similarly acknowledged the advantages of technology in easing their daily routines. Participant P8 noted, “Smart attendance equipment simplifies the manual attendance process, saving valuable time for parents and teachers”. This sentiment reflects the growing reliance on technology to automate time-intensive tasks and improve overall operational efficiency.

Teachers also emphasized how technology has streamlined administrative processes, reducing the complexity of manual operations. Participant T8 explained, “Before utilizing technology, the health staff had to manually confirm attendance for each class through calls, which was complex. Now, technology allows attendance figures to be directly uploaded, reducing the workload for healthcare staff and improving speed and efficiency”. The ability to upload attendance data in real-time not only reduces the burden on healthcare personnel but also enhances the accuracy and speed of data processing, allowing more time for critical tasks.

Parents have particularly appreciated the integration of attendance data with mobile apps, which provide real-time updates on their children’s entry and exit times. Participant P6 highlighted this convenience, explaining how the smart attendance device connects to an app, enabling them to monitor their children’s schedules seamlessly. This transparency fosters better communication between parents and teachers, ensuring a collaborative approach to managing children’s daily routines.

The integration of smart attendance systems and health monitoring robots has significantly modernized traditional healthcare practices in kindergarten settings. By automating repetitive tasks and providing comprehensive data, these technologies enhance the efficiency and accuracy of operations.

#### 3.3.2. Positive Outcomes in Stakeholder Collaboration

The introduction of digital tools has also strengthened communication between stakeholders, fostering a more collaborative environment in kindergarten settings. Real-time data sharing has improved the transparency of operations, allowing parents, teachers, and healthcare staff to work together more effectively.

Parents benefit from direct access to attendance and health information through digital platforms. This connectivity ensures they remain informed about their children’s schedules and well-being. Participant P6 emphasized how the integration of the smart attendance system with mobile apps creates a seamless experience, allowing for better coordination between parents and educators.

Teachers and healthcare personnel have similarly benefited from enhanced communication pathways enabled by real-time data sharing. This is particularly evident in scenarios requiring tailored support for children with specific needs. For example, attendance data allows nutritionists and chefs to prepare individualized meals for children with allergies or dietary restrictions. Participant T1 elaborated:

Smart attendance equipment expeditiously informs us of the attendance numbers, including those of children with special needs. This is crucial, as children with allergies or specific dietary requirements necessitate distinct meals. Nutritionists and chefs utilize the data collected by the smart attendance device, facilitating the preparation of tailored recipes for individual children.

Teachers also use real-time data to align their efforts with other staff members, ensuring consistency and precision in their daily operations. The ability to access and share attendance and health metrics creates a cohesive and supportive environment, enabling educators to deliver more personalized care to children.

This enhanced connectivity reflects a broader trend of using technology to bridge communication gaps, build a cohesive community, and align stakeholders’ efforts in kindergarten settings. By streamlining information flow, these technologies foster teamwork and improve the overall care and development of children. Participant P8 noted the broader implications of these advancements: “The introduction of technology has made our communication more efficient and transparent. It’s easier to coordinate with teachers and support staff when we have the same real-time information”.

The integration of healthcare technologies has not only modernized traditional practices but has also created new pathways for collaboration, demonstrating how digital tools can strengthen relationships and align efforts within early childhood education settings.

#### 3.3.3. Emerging Needs

Despite its benefits, the current use of technology in kindergartens reveals gaps in data accessibility and integration. Of the 18 parents interviewed, 55% expressed a desire for access to their children’s health information, such as body temperature, height, and weight, to foster stronger collaboration with schools. Participant P16 emphasized, “We hope that all kinds of information about young children can be concentrated on one app or platform, as long as the classification is clear”.

While increased transparency can empower parents and support more holistic child development, it also raises concerns about data privacy and security. Participant T5 proposed developing a centralized platform to integrate health, attendance, and educational data, ensuring seamless communication among departments while safeguarding sensitive information. This vision aligns with the broader objective of creating a unified, secure system for data sharing, enabling greater collaboration without compromising privacy.

### 3.4. Data Sharing and Integration

***Bridging Home–School Communication.*** One of the most significant concerns among parents is the limited accessibility of health information collected by digital devices. Of the 18 parents interviewed, 10 (55%) expressed a strong desire for access to their children’s health metrics, such as body temperature, height, and weight. This demand aligns with the evolving emphasis on home–school collaboration, where parents are more actively involved in their children’s well-being [48]. Participant P16 articulated, “We hope all kinds of information about young children can be concentrated on one app or platform, as long as the classification is clear”.

However, sharing sensitive health information raises privacy and security concerns. Kindergartens need to balance transparency with privacy, necessitating well-defined policies and security measures for data management and information safeguarding, ensuring a responsible and secure data-sharing environment.

***Expanding Technology Use Beyond Healthcare in Schools.*** The integration of technology in kindergartens has proven valuable in streamlining healthcare-related tasks, but its potential extends well beyond these applications. Teachers and parents envision broader uses for health data to enhance both educational strategies and administrative processes, leveraging the synergy between healthcare and education for a more integrated approach to child development.

***Health Data Supporting Educational Strategies.*** Teachers see significant potential for health data collected by the morning inspection robot to inform educational initiatives. Participant T3 highlighted this application: “Through the morning inspection robot, we can assess the obesity rate across the entire kindergarten. This insight prompts consideration of developing educational topics and implementing teaching strategies, such as enhanced exercise activities, to address and mitigate childhood obesity”.

This data-driven approach emphasizes the role of health metrics in identifying trends, enabling educators to design targeted programs that address specific needs. For example, obesity data could guide the development of physical education programs or school-wide campaigns promoting healthy lifestyles. Participant T3’s perspective underscores the untapped potential of integrating health data into teaching contexts, fostering a more holistic view of child development.

Participant T2 also emphasized the value of technology in streamlining nutritional analysis, an area traditionally managed through labor-intensive manual processes:

In the past, nutritional analysis required manual efforts by healthcare staff, necessitating monthly data collection and subsequent aggregation for analysis. Presently, with technological support, healthcare personnel simply input each child’s recipes and food intake weekly, leveraging big data for precise individual analyses.

This efficiency not only reduces the workload for healthcare staff but also provides educators with actionable insights to tailor health education and dietary practices to the needs of individual children.

***Centralized Platforms for Data Integration*.** Participants also identified the need for a centralized platform to integrate health data with broader school management processes. Participant T5 proposed this solution: “An integrated information platform would enhance interdepartmental connectivity, enabling the seamless sharing of information among various departments. This would optimize the efficiency of educational endeavors within the school”.

Such a system could streamline communication and decision-making by providing all relevant departments with real-time access to critical information. For example, attendance and health data could inform both healthcare initiatives and educational planning, creating a more cohesive and efficient organizational structure.

Parents echoed this desire for a centralized approach. Participant P16 remarked, “We hope that all kinds of information about young children can be concentrated on one app or platform, as long as the classification is clear”. This preference for unified data management reflects a broader trend toward integrated systems that simplify information access for both educators and families.

***Transforming Administrative Processes*.** By expanding the use of health data and integrating it into centralized platforms, kindergartens have the potential to transform not only healthcare practices but also educational and administrative processes. These advancements could improve the overall functionality of early childhood education systems by enhancing efficiency, promoting collaboration across departments, and supporting personalized care and learning strategies.

Participant T5 summarized the broader implications of this approach:

This platform ideally should facilitate interdepartmental connectivity, enabling the seamless sharing of information among various departments. Such integration would enhance the organizational structure of the entire kindergarten, resulting in a more grid-based management system, thereby optimizing the efficiency of educational endeavors within the school.

By leveraging health data for both educational and administrative purposes, kindergartens can create a more holistic, efficient, and child-centered approach to early education, benefiting stakeholders across the board.

***Need for In-Depth Data Analysis.*** The current implementation of data usage in kindergarten settings remains rudimentary, neglecting the comprehensive potential of healthcare data in facilitating informed decision-making. Participant T1 highlighted the current limitations, stating, “We use health data collected through technology to establish a health file for each child, gaining insights into the overall health of the kindergarten and understanding the developmental progress of individual children”. However, the data are often used only for basic record-keeping, with little effort to leverage it for deeper insights or strategic decision-making.

Participant P9, the principal of the kindergarten, emphasized the need for more sophisticated analysis:

The kindergarten attendance equipment provides a quick count of children’s attendance, but the analysis often stops at the surface level. Many teachers only focus on getting the number of children in attendance and rarely think further. For example, where a decrease in attendance could signal underlying issues like seasonal changes or a societal influenza outbreak.

These findings suggest a pressing need for advanced training and tools to help educators interpret and apply health data effectively. By expanding the scope of data analysis, kindergartens can implement more informed and proactive interventions to enhance children’s well-being.

## 4. Discussion and Implications

The integration of healthcare technologies, such as smart attendance devices and morning inspection robots, represents a significant advancement in early childhood education. These innovations address critical challenges, such as insufficient healthcare staff, and improve operational efficiency in kindergartens. Furthermore, they foster the development of digital health literacy among children, teachers, and parents, equipping stakeholders with vital skills to navigate a technology-driven world [49,50]. As children grow up in increasingly digital environments, their early interactions with such systems prepare them to handle potential risks and opportunities [51].

Although these technologies demonstrate promising benefits, their integration also reveals areas that require further attention. The following discussion outlines both the successes achieved and recommendations for future advancements.

### 4.1. Areas for Improvement and Future Directions

#### 4.1.1. Child-Centric Design and Accessibility

Technologies in early education must prioritize the preferences and needs of young children. Devices should be adapted to accommodate children’s height and ergonomic requirements to ensure accessibility for all users. Participants emphasized the importance of integrating child-friendly elements, such as familiar characters and interactive designs, to make technology more appealing and engaging [52].

Additionally, user-friendly interfaces with clear instructions are essential for ensuring accessibility to a diverse range of stakeholders, including parents with varying levels of technical literacy. To further enhance accessibility, developers could incorporate adaptive interfaces, such as simplified navigation for children or larger text and visual cues for older adults. This inclusive design not only broadens adoption but also enhances the overall user experience by fostering confidence and autonomy in system interaction.

#### 4.1.2. Non-Verbal Communication Features

Observations revealed that many children prefer non-verbal modes of communication, such as eye contact and gestures, when interacting with technology. Future updates could incorporate features that recognize and respond to these cues, such as gesture-based controls or visual prompts. These features would align more closely with children’s natural communication tendencies, creating a more engaging and accessible experience for young users.

#### 4.1.3. Advanced Health Data Utilization

The potential of health data extends beyond its current applications in kindergarten settings. Sophisticated data analysis can provide valuable insights into trends such as obesity rates or attendance patterns, enabling targeted health interventions and educational strategies. For example, health data from the morning inspection robot could inform school-wide initiatives to promote healthy lifestyles and physical activity.

However, expanded use of health data necessitates robust data governance policies to address privacy and security concerns. Schools must establish clear guidelines on data collection, storage, and access while investing in secure infrastructure to protect sensitive information. Implementing advanced encryption techniques and access control mechanisms can further safeguard health data, ensuring only authorized personnel have access to sensitive records. Additionally, transparent policies and adherence to legal and ethical standards are critical for maintaining trust among stakeholders. Regular data audits and compliance checks with relevant legal frameworks, such as data protection laws and child privacy regulations, are essential for identifying vulnerabilities and maintaining data security.

#### 4.1.4. Infrastructure Development and Teacher Preparedness

Reliable digital infrastructure, including strong network connectivity, is fundamental for the successful integration of healthcare technology. Participants noted that network disruptions often hindered device functionality, emphasizing the need for consistent and robust infrastructure.

Teacher preparedness is equally important. Efficient management of tasks such as uploading children’s data to systems requires comprehensive training programs. Providing educators with the tools and knowledge to effectively operate these technologies not only enhances functionality but also empowers teachers to explore creative applications of the systems.

## 5. Conclusions

The integration of healthcare technology in kindergarten settings represents a transformative step toward creating safer and more efficient learning environments. These tools address operational challenges, improve efficiency for healthcare staff, and equip children and educators with essential skills for the digital age.

Future advancements should focus on child-centric designs, non-verbal communication features, advanced health data utilization, and robust data governance to ensure privacy and security. Additionally, investments in infrastructure and teacher training are crucial to fostering a supportive environment for these technologies. By addressing these priorities, healthcare technologies can fully realize their potential, contributing to holistic child development and collaborative early education practices.

While this study focuses on kindergarten health and safety routines, future research should investigate the scalability and sustainability of these innovations across a broader range of educational contexts, including different age groups and school settings. Incorporating a wider array of quantitative and qualitative methodologies provides a more comprehensive understanding of their effects. Furthermore, longitudinal and intervention studies could offer valuable insights into the long-term implications of technology integration on child development, teacher practices, and overall school operations. Expanding the application of healthcare technologies to diverse educational environments will further illuminate their adaptability and impact for student development and institutional processes.

## Figures and Tables

**Table 1:** Demographic information of teachers and principals.

Number	Gender	Role	Years of Experience
T1	Female	Teacher	15 years
T2	Female	Teacher	20 years
T3	Female	Teacher	4 years
T4	Female	Teacher	8 years
T5	Female	Teacher	12 years
T6	Female	Teacher	19 years
T7	Female	Teacher	3 years
T8	Female	Teacher	1 year
T9	Female	Principal	9 years
T10	Female	Principal	14 years
T11	Female	Principal	20 years

Notes. T = Teacher.

**Table 2:** Demographic information of parents.

Number	Relation to Child	Age Group
P1	Mother	30s
P2	Mother	20s
P3	Mother	20s
P4	Mother	30s
P5	Mother	30s
P6	Mother	30s
P7	Grandfather	60s
P8	Mother	20s
P9	Grandfather	60s
P10	Grandmother	60s
P11	Mother	20s
P12	Father	30s
P13	Mother	30s
P14	Mother	20s
P15	Mother	20s
P16	Grandfather	60s
P17	Grandmother	50s
P18	Grandmother	50s

Notes. P = Parent; 20s = ages 20–29; 30s = ages 30–39; 50s = ages 50–59; 60s = ages 60–69.

**Table 3:** Demographic information of children.

Number	Gender	Age
C1	Female	5 years old
C2	Female	6 years old
C3	Male	6 years old
C4	Male	5 years old
C5	Male	6 years old
C6	Male	5 years old
C7	Male	5 years old
C8	Male	5 years old
C9	Male	5 years old
C10	Female	5 years old
C11	Female	6 years old

Notes. C = Child.

**Table 4:** Examples of inductive coding.

Step	Content	
Data extract	Child (C2): I like robots because there are many buttons and QR codes on the robots, which are fun.	Child (C5): The morning inspection robot looks a bit scary. It would be great if it could resemble Elsa and engage in interactive games with me.
Refinement	Positive perception of technical features	Negative perception of technical features
Advanced Theme	Preferences for the technical appearance of robots	

**Table 5:** Coding scheme.

First Level Code	Second Level Code	Third Level Code
Technology in Kindergarten Health and Safety Practices	Smart Attendance System	Technology and Human Interactions
		Interaction Dynamics
	Morning Inspection Robot	Technology and Human Interactions
		Interaction Dynamics
Barriers to Effective Technology Integration	Infrastructure Challenges	Technical Limitations
		Network Connectivity Issues
		Inefficient Processes
	Challenges in User Engagement and System Design	Technology Needs to be User-Friendly
		Training and Support for Effective Use
Impacts of Technology on Kindergarten Practices	Improved Operational Efficiency	
	Positive Outcomes in Stakeholder Collaboration	Strengthening Communication Between Parties
	Emerging Needs	Data Sharing and Integration
		Need For In-Depth Data Analysis

**Table 6:** Illustration of parents as facilitators in assisting children with technology.

Screenshot of Video 1	Content
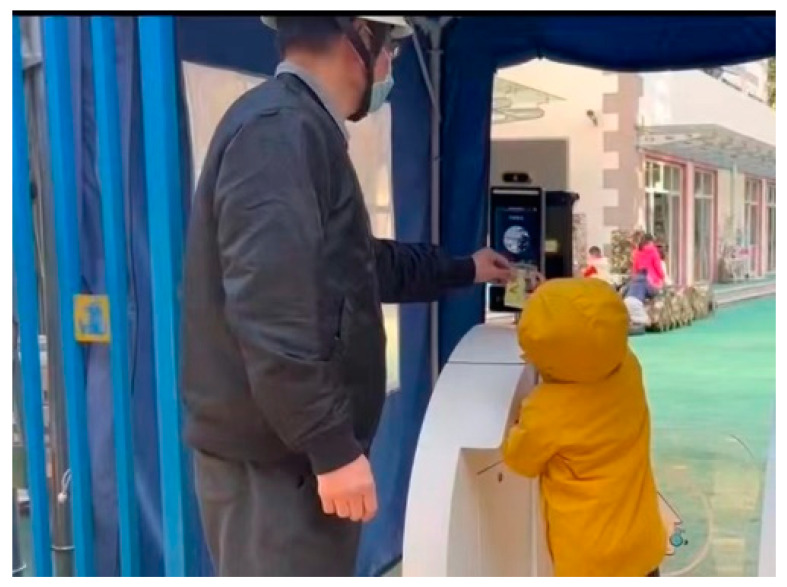 Screenshot of parent as “Facilitator” in assisting child with technology in video 1	A child attempted to scan the QR code on the designated area of the screen. Due to misalignment, the device failed to recognize the child’s input, and no prompts were provided to guide the child in correcting the issue. Eventually, the parent intervened, instructing the child to adjust the movement upwards and backward, collaboratively working to complete the operation.

**Table 7:** Frequency table for the use of the smart attendance device.

Classification	Code	N
Roles of technology	Independent Actor	19
	Facilitator	0
	Responsive Entity	0
Roles of parents	Observer	19
	Facilitator	2
	Influencer	0
Roles of children	Observer	1
	Influencer	0
Interaction Dynamics	Active Communication	1
	Passive Communication	1
	Refusal to Communicate	0
	No Communication	17
	Autonomous Use of Technology	17
	Guided Use of Technology	2
	Refusal to Use Technology	0

**Table 8:** Example of children discussing the morning inspection robot as they line up.

Screenshot in Video 3	Content
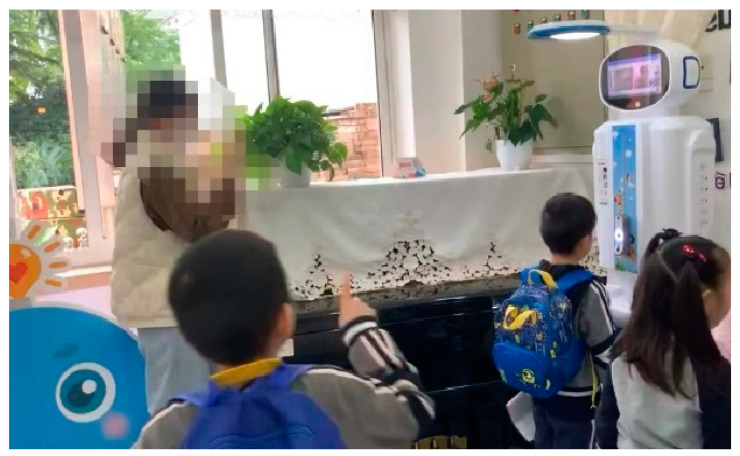 Screenshot of children discussing the morning inspection robot as they line up in video 3	The child enthusiastically called out to his companion multiple times and joyfully pointed towards the robot, indicating that he was about to use the morning inspection robot and inviting his companions to follow him.

**Table 9:** Frequency table for the use of morning inspection robot.

Classification	Code	N
Roles of Technology	Independent Actor	21
	Facilitator	19
	Responsive Entity	0
	Interactor	18
Roles of Children	Observer	39
	Influencer	0
	Interactor	18
Interaction Dynamics	Active Communication	1
	Passive Communication	4
	Refusal to Communicate	12
	No Communication	18
	Eye Contact with the Robot	14
	Utilize or Explore the Robot Autonomously	12
	Utilize the Robot Under Guidance	13
	Refusal to Utilize the Robot	10

## Data Availability

The data supporting the findings of this study, including video recording and interview transcripts, are not publicly available to ensure the privacy and confidentiality of the participants. Aggregated and anonymized data may be shared upon reasonable request to the corresponding author, subject to ethical approval and participant confidentiality agreements.
